# Lost and Found: A Case of a Misplaced Foreign Body in Childhood causing Cholesteatoma as an Adult

**DOI:** 10.51894/001c.144477

**Published:** 2025-10-01

**Authors:** Vaibhav Kadam

**Affiliations:** 1 Department of Otolaryngology McLaren Oakland

## 88

### INTRODUCTION

Cholesteatoma is a benign but locally destructive pathology composed of keratinizing squamous epithelium with an accumulation of keratin debris. Middle ear foreign bodies have been implicated in cholesteatoma formation by inducing a chronic inflammatory reaction. Here we describe a unique case of a nasal foreign body leading to chronic eustachian tube dysfunction with subsequent cholesteatoma formation.

### CASE PRESENTATION

A 65-year-old female presented to the neuro-otology clinic for chronic left-sided otorrhea for the previous 3 months. The exam was significant for otorrhea and pars flaccida retraction. A computed tomography scan of the temporal bones demonstrated complete opacification of the left middle ear cavity and mastoid air cells. An incidental finding of a circular density with surrounding calcifications was present in the left posterior nasal cavity. Decision was made to proceed with left tympanomastoidectomy with ossicular chain reconstruction, left eustachian tube dilation, and nasal endoscopy with removal of the foreign body. The left nasal endoscopy found a button foreign body with over 60 years of surrounding calcifications and irritation of the surrounding structures. During the tympanomastoidectomy, the cholesteatoma sac was carefully dissected along with the ossicles and replaced with a total ossicular chain prosthesis. During the postoperative visit, the patient noted she has always had difficulty breathing from her left nostril. The post-operative audiogram demonstrated an approximate 40-decibel improvement for the left ear.

### DISCUSSION

A variety of theories have been proposed for the pathogenesis of acquired cholesteatoma. In our case, the formation of cholesteatoma was a result of poor middle ear space aeration from chronic eustachian tube dysfunction leading to pars flaccida retraction. This was a culmination from chronic inflammation from a long-standing button foreign body in the left posterior nasal cavity. The obstruction of eustachian tube opening led to chronic negative pressure of the middle ear. It is essential to conduct a complete and thorough history and physical exam with supplemental imaging to help identify unusual causes of cholesteatoma.

